# Vision and Foraging in Cormorants: More like Herons than Hawks?

**DOI:** 10.1371/journal.pone.0000639

**Published:** 2007-07-25

**Authors:** Craig R. White, Norman Day, Patrick J. Butler, Graham R. Martin

**Affiliations:** Centre for Ornithology, School of Biosciences, The University of Birmingham, Birmingham, United Kingdom; Zoological Society of London, United Kingdom

## Abstract

**Background:**

Great cormorants (*Phalacrocorax carbo* L.) show the highest known foraging yield for a marine predator and they are often perceived to be in conflict with human economic interests. They are generally regarded as visually-guided, pursuit-dive foragers, so it would be expected that cormorants have excellent vision much like aerial predators, such as hawks which detect and pursue prey from a distance. Indeed cormorant eyes appear to show some specific adaptations to the amphibious life style. They are reported to have a highly pliable lens and powerful intraocular muscles which are thought to accommodate for the loss of corneal refractive power that accompanies immersion and ensures a well focussed image on the retina. However, nothing is known of the visual performance of these birds and how this might influence their prey capture technique.

**Methodology/Principal Findings:**

We measured the aquatic visual acuity of great cormorants under a range of viewing conditions (illuminance, target contrast, viewing distance) and found it to be unexpectedly poor. Cormorant visual acuity under a range of viewing conditions is in fact comparable to unaided humans under water, and very inferior to that of aerial predators. We present a prey detectability model based upon the known acuity of cormorants at different illuminances, target contrasts and viewing distances. This shows that cormorants are able to detect individual prey only at close range (less than 1 m).

**Conclusions/Significance:**

We conclude that cormorants are not the aquatic equivalent of hawks. Their efficient hunting involves the use of specialised foraging techniques which employ brief short-distance pursuit and/or rapid neck extension to capture prey that is visually detected or flushed only at short range. This technique appears to be driven proximately by the cormorant's limited visual capacities, and is analogous to the foraging techniques employed by herons.

## Introduction

Pursuit–dive foraging (taking prey from the water column or from substrata at depth) is widespread among birds (c.150 species from seven Orders). Although key aspects of the diet and foraging ecology of many of these species are known, little information is available regarding how these birds actually detect prey and what factors constrain their diving behaviour. Amphibious behaviour presents major sensory problems to birds, because of the markedly different properties of air and water. The optical requirements for aquatic vision are fundamentally different from those in air, because underwater light environments differ from aerial environments in spectral composition, luminance and turbidity [1], [2]. Furthermore, upon entering water, eyes of terrestrial vertebrates experience the loss of corneal refractive power and to retain a sharp retinal image this loss must be compensated for by changes in the lens [3]. This loss of corneal refractive power also results in the reduction in the sizes of visual fields, alteration of visual field topography and reduction in the brightness of the retinal image [4], [5].

Great cormorants (*Phalacrocorax carbo*: Phalacrocoracidae) are generally regarded as visually-guided, pursuit-dive foragers, which have the highest known foraging yield for a marine predator [6] and very seldom injure fish without catching them [7]. It may therefore be predicted that cormorants have excellent vision much like aerial predators, such as hawks which detect and pursue prey from a distance. Great Cormorants are widely distributed with resident populations from temperate latitudes in the southern hemisphere (e.g. New Zealand; 45°S) through the tropics to as far north as Greenland (70°N) in the northern hemisphere [8], [9]. Throughout this range they are often perceived as being in conflict with human fisheries interests [10]. They exploit fish resources in coastal waters, freshwater lakes and rivers. Cormorants exhibit a range of solitary and social foraging behaviours and group foraging appears to be particularly effective in highly turbid waters [25]. Individuals are known to dive, presumably in pursuit of prey, at night in the middle of winter at high latitudes [11]. They are known to forage on both pelagic and benthic fish species [7], [10]. Cormorant populations in Greenland and Iceland are known to forage mainly on sculpins (*Myoxocephalus*) [6], [12], which are a group of cryptically coloured benthic fish with a disruptive outline pattern that may have evolved in response to avian predation pressure [13]. Given their ability to prey upon pelagic and cryptic benthic prey, and a high capacity to accommodate their eye's optical system to compensate for the loss of corneal refractive power upon immersion [3], [14]–[16], it is reasonable to expect that cormorants have a visual system well adapted to function in water and that, as in aerial predatory birds, vision is the primary sense that guides their foraging. Indeed, cormorant eyes appear to show some specific adaptations to the amphibious life style. Thus, they were reported to have a highly pliable lens whose curvature is driven by powerful intraocular muscles [14]–[16] and this is thought to accommodate for the loss of corneal refractive power that accompanies immersion and ensures a well focussed image on the retina [3].

However, demonstration of an anatomical capacity to accommodate visually for the changes occurring upon immersion provides little indication of the visual information available to a cormorant when foraging underwater. To develop an improved picture of what a diving cormorant sees when foraging, we used established psychophysical visual discrimination training techniques to determine the upper limits of cormorant visual acuity under water. We determined the visual acuity thresholds of free-swimming cormorants under a range of viewing conditions that mimicked those experienced in clear waters at different naturally occurring light levels when viewing targets of different contrasts and at different viewing distances. We then used these data to model the appearance of a typical target fish viewed by cormorants at a range of distances, target contrasts, and illuminations representative of those encountered by naturally foraging birds.

## Results

**Figure 1:**
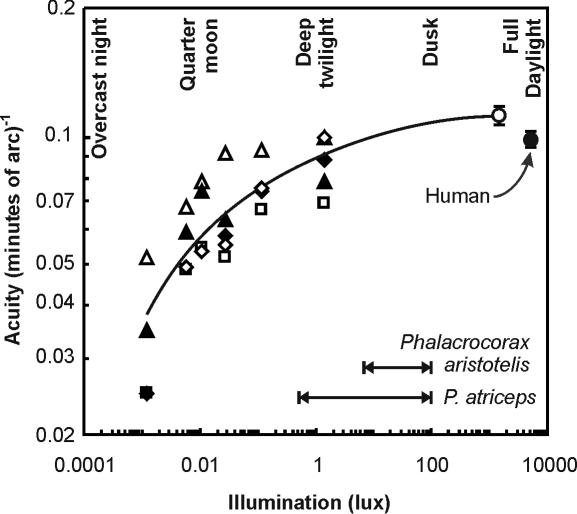
Effect of ambient illumination (lux) on the visual acuity of five great cormorants *Phalacrocorax carbo*. Visual acuity is expressed as the reciprocal of minutes of arc. The relationship is significant: log(acuity) = −0.00168 log(illumination)^2^ + 0.0125 log(illumination) + 0.0889. Symbols represent individual birds: ▴, ▵, ⧫, ◊, □. Mean values±SEM: 0.034±0.006, 0.055±0.004, 0.063±0.005, 0.064±0.007, 0.077±0.006, 0.087±0.006 for illuminations of 0.0012, 0.0058, 0.011, 0.028, 0.11, and 1.4 lux, respectively. ○ = mean data±SEM for five great cormorants determined by Strod et al [17]; • = mean aquatic visual acuity threshold for unaided humans [19]. The range of mean illumination encountered during the bottom phase of dives is shown for European shags *Phalacrocorax aristotelis* and blue-eyed shags *Phalacrocorax atriceps* [18], as are the illumination levels equivalent to those received at the earth's surface from natural sources between full daylight and an overcast night.

**Figure 2 pone-0000639-g002:**
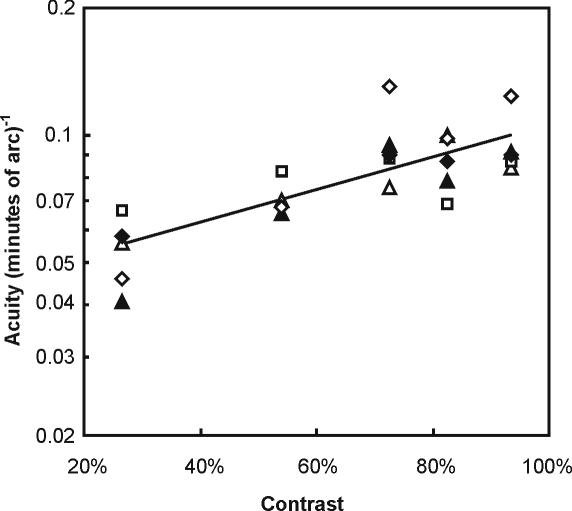
Effect of contrast on visual acuity of five great cormorants. The relationship is significant: log(acuity) = −1.36+0.38 (contrast). Symbols represent individual birds: ▴, ▵, ⧫, ◊, □. Mean values±SEM: 0.054±0.005, 0.071±0.004, 0.096±0.009, 0.087±0.006, 0.095±0.007 (minutes of arc)^−1^ for contrast of 27, 54, 72, 82, and 93%, respectively.

**Figure 3:**
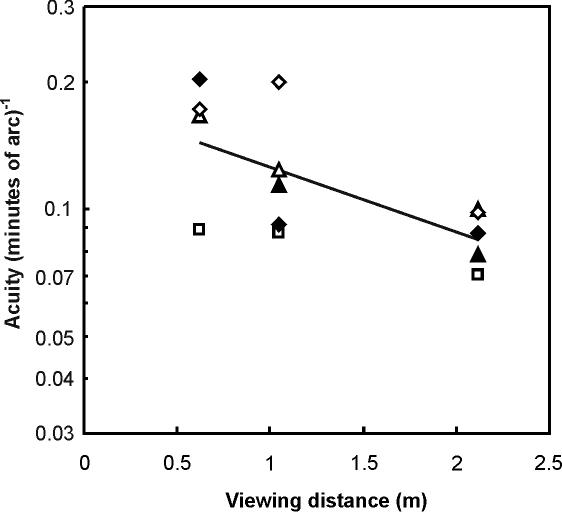
Effect of viewing distance on visual acuity of five great cormorants. The relationship is significant: log(acuity) = −0.751–0.151 (viewing distance). Symbols represent individual birds: ▴, ▵, ⧫, ◊, □. Mean values±SEM: 0.15±0.03, 0.12±0.03, and 0.087±0.006 (minutes of arc)^−1^ for viewing distances of 0.62, 1.05, and 2.12 m, respectively

A total of 9673 discrimination trials were scored, and these were preceded by and interspersed with 11853 training trials which maintained 100% correct discrimination performance for high contrast, low spatial frequency stimuli. The number of trials per session varied significantly between birds (ANOVA, F_4,252_ = 30.4, p<0.0001) and ranged from 15.5±0.1 (SEM) to 26.3±0.2. Visual acuity was significantly effected by target illumination (Fig. 1, F_5,18_ = 39.0, p<0.0001), target contrast (Fig. 2, F_4,15_ = 10.2, p = 0.0003), and viewing distance (Fig. 3, F_1,8_ = 16.6, p = 0.003). Visual acuity was positively related to target illumination and contrast, and negatively related to viewing distances (Figs 1–3).

## Discussion

Our overall conclusion is that the ability of cormorants to resolve visual detail in water is poor, and far below that predicted by analogy with the vision of predatory birds that take prey in aerial pursuit. Thus, the mean visual acuity of great cormorants for targets with high (82%) contrast at an illumination equivalent to that of twilight (1.4 lux) equalled 11.8±0.8 [SEM] minutes of arc. Acuity improved at higher (day-time) levels of illumination [17], but the difference was slight, and acuity was low at the levels of illumination (ca 0.5 to 100 lux) that cormorants are known to encounter during natural dives [18]. To provide a perspective on this relatively poor visual acuity in cormorants it should be noted that the cormorants' highest visual performance is only equal to that of unaided humans in water [19], and approximately 60 times lower than that of visually-guided terrestrial avian predators, such as eagles, whose acuity threshold lies between 0.2–0.8 min of arc [20]–[23]. This is a surprising result for a predator that exhibits high foraging efficiency [6] and is assumed to be visually guided [8].

### A model of prey detectability

**Figure 4:**
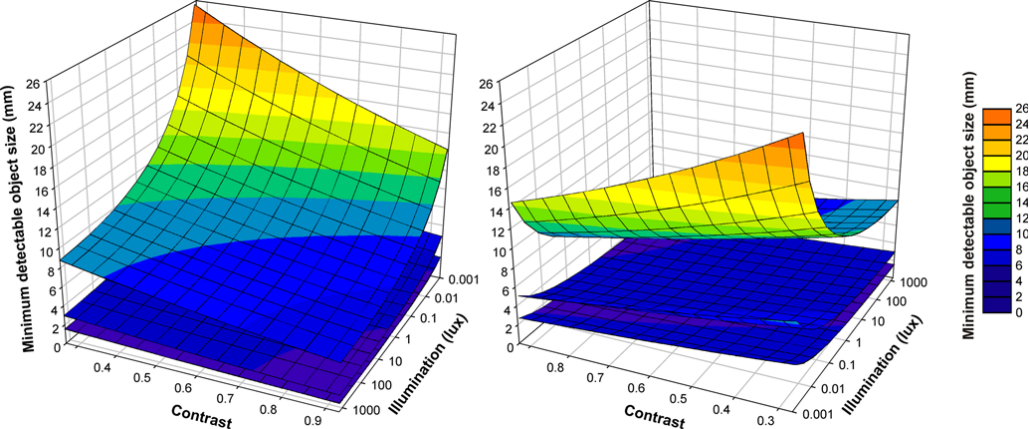
Visual acuity surfaces of great cormorants describing the effects of contrast, illumination and viewing distance. Three surfaces are presented, corresponding with viewing distances of 2.12 m (upper surface), 1.05 m (middle surface) and 0.63 m (lower surface). Visual acuity is expressed as the minimum width of a detectable object (mm).

**Figure 5:**
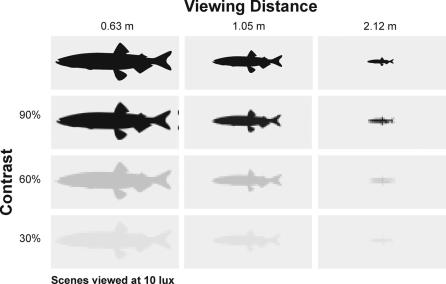
Prey detectability model for a great cormorant based upon the data of Fig. 4 demonstrating the effects of contrast and viewing distance. The model is based upon a great cormorant foraging on a capelin (*Mallotus villosus*, 10 cm TL) type fish at an ambient illumination of 10 lux, which has a contrast of 90, 60 and 30% viewed from a distance of 0.63, 1.05 or 2.12 m. Each frame depicts a scene with an angular width of 10°. Scenes were generated by determining the angular resolution appropriate to each set of conditions from Fig. 4, and appropriately downsampling the high resolution images in the upper row.

**Figure 6:**
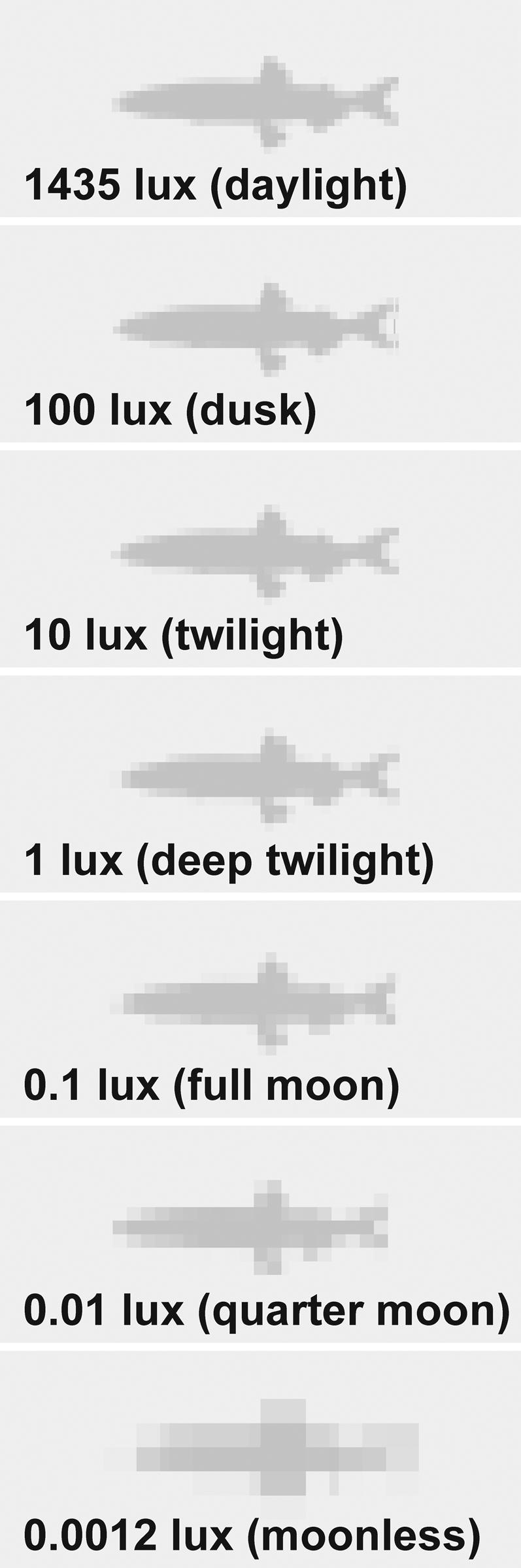
Prey detectability model for a great cormorant demonstrating the effect of illumination. The model is based upon a great cormorant foraging on a capelin (*Mallotus villosus*, 10 cm TL) of 60% contrast viewed at a distance of 1.05 m over a range of ambient illumination levels equivalent to those received at the earth's surface from natural sources between daylight to a moonless night. These span the range of target light levels used in this series of experiments and span the range of ambient light levels that are known to be encountered by cormorants during natural dives [18] (ca 0.5 to 100 lux). Each frame depicts a scene with an angular width of 10°.

To explore the consequences of the cormorants' poor visual resolution we have used our acuity data to model prey detectability under a range of viewing conditions. We used curves fitted to our acuity-illumination (Fig. 1), acuity-contrast (Fig. 2), and acuity-viewing distance (Fig. 3) functions to describe a series of “threshold acuity surfaces” which relate acuity to target contrast and illumination, for each viewing distance (Fig. 4). This encapsulates within a single figure the ways in which acuity is influenced by a range of important parameters that describe the visual tasks encountered by foraging cormorants. From this we have been able to model visual prey detectability in cormorants under a range of viewing conditions. Figs 5 and 6 show two examples from this modelling using a prey item of a size (10 cm total length) commonly taken by cormorants [24], [25]. Even for a prey item of this size detectability is low at all but the highest target contrasts, light levels and short viewing distances. This raises a number of important questions concerning the foraging techniques of cormorants and the predator-prey interactions which underlie them.

We have modelled prey detectability in cormorants using a relatively high contrast prey item stimulus based upon a pelagic fish which would be taken from a water column with low turbidity. This presents probably the simplest foraging situation for a cormorant and therefore encapsulates what is likely to be the maximum visual performance when foraging. Thus, acuity will decline further with increasing turbidity [17], and high-contrast pelagic prey are not typical items for cormorants. Potential prey animals in benthic nearshore habitats have evolved to evade detection through the use of both masquerade (i.e. resembling an object that is not normally eaten) and eucrypsis (i.e. resembling the background) strategies [26]. In the euphotic pelagic zone, prey species have evolved transparency or reflectivity, with the latter often accompanied by countershading [26]. The actual visual prey detectability in cormorants is therefore likely to be far lower than the upper limits modelled in Figs. 5 and 6 based upon simple contrast parameters. The modelled prey detectability strongly suggest that the foraging strategies of cormorants are likely to be constrained by their poor aquatic visual acuity. We propose that foraging cormorants must adopt a range of behavioural strategies to overcome the limits of their vision.

### Foraging strategies of cormorants

Under certain conditions cormorants are known to forage co-operatively. Thus, in turbid conditions, where acuity will be further reduced compared with the acuity thresholds reported here [17], cormorants may use mass fishing techniques to drive fish to relatively clear surface waters where they are more likely to be detected when seen from below in silhouette against the downwelling light [27]. However, cormorants more typically forage alone, often in turbid conditions at depths greater than 10m where light penetration is low, and sometimes at night [11], and it has been suggested that cormorants might locate prey by touch using the bill [28]. Tactile detection is thought to be successful only when prey density is sufficiently high, when fish are relatively immobile (as in the case in hibernating aggregations), or both [28]. We propose that these kinds of specialised behavioural strategies play an important role in all cormorant foraging.

We propose that these observations on foraging behaviour, together with our threshold acuity data (Fig. 4), suggest that cormorants do not, and cannot, detect and pursue prey underwater in a way that is analogous to that of predatory birds, such as hawks, in air. Indeed, images from bird-borne cameras on the congeneric European shag *Phalacrocorax aristotelis* show that foraging typically occurs on the seabed rather than in the water column [29], and high underwater swimming speeds indicative of prey pursuits are very rare in great cormorants [30]. Cormorants must either detect prey visually but only at very short distances, or use a prey-flushing strategy [31] that forces prey to make an escape response. In either case it would seem inappropriate to describe cormorants as pursuit foragers.

### Cormorant foraging: more like herons than hawks?

The cormorants' ability to strike rapidly at near prey employing rapid extension of their long necks whilst virtually anchored by their body mass and large webbed feet, might be a way to capture food without an energetically expensive pursuit [30], [32], This technique may be key to this species' ability to forage efficiently in a wide range of aquatic environments and on different types of prey whose combination would appear to pose a wide range of perceptual challenges. Thus we propose that the foraging success of great cormorants does not lay in particular adaptations of its vision to resolve fine spatial detail within different aquatic environments, but in the evolution of foraging techniques that operate within the constraints of its vision. These foraging techniques, are analogous to those employed by herons (Ardeidae) that use single-strike lunging to take evasive prey [33]. We conclude that although cormorants are highly efficient predators their aquatic foraging technique is more like that of a lunging heron than an aerial pursuing hawk

## Materials and Methods

**Figure 7:**
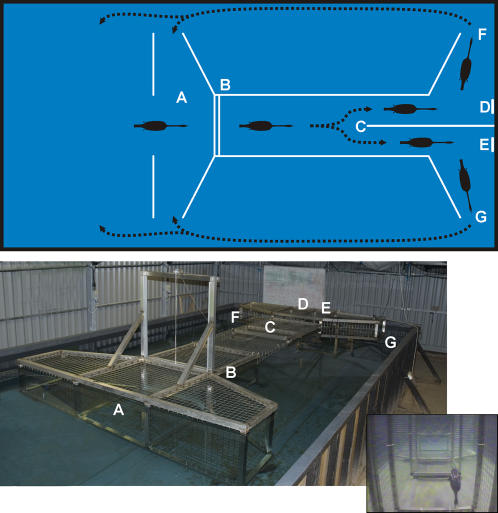
Stylised scale plan view of the tank (8 m×4 m) and swimway apparatus showing the typical paths followed by a cormorant during a discrimination trial (Top) and photograph of the tank and swimway apparatus (bottom). Birds began a trial in the starting area (A). When the gate (B) was raised by the experimenter, signalling the start of a trial, the birds entered the swimway and performed the simultaneous discrimination at a point (C) a known distance from the stimuli (D and E). If the birds approached the correct stimulus they were provided with a fish reward (a single sprat). If they approached the incorrect stimulus, they received no reward. At the end of each trial, the birds exited the swimway at F or G, and returned to the starting position (A) to await the start of a new trial. Note that the stimuli are submerged beneath D and E in the photograph. Inset shows a single frame captured from a video of a bird swimming though the swimway, just prior to the choice point (C). See Movie S1 for the full video sequence.

Five great cormorants (*Phalacrocorax carbo*) were trained using positive reinforcement operant conditioning to conduct a simultaneous visual discrimination [17], [34] between pairs of horizontal and vertically orientated gratings which were presented at the end of a stainless steel swimway in a random sequence (Fig. 7). The gratings were printed on acetate sheets and trans-illuminated by light from a tungsten source. The level of trans-illumination was controlled by neutral density filters and measured in situ at the gratings. The level of grating contrast was controlled by the density of printing and measured in situ with an Ocean Optics 80X Optometer. Stimulus contrast was defined as (I_max_−I_min_)/(I_max_+I_min_)×100%. The whole swimway and stimulus presentation apparatus was submerged in a 1 m deep 8×4 m tank filled with continuously replenished freshwater, which was housed in a light proof building. This ensured high water clarity throughout the experiments. Turbidity was monitored periodically with a portable Hach 2100P turbidimeter, and remained below 1 NTU (nephlometric turbidity unit). The building was illuminated by banks of fluorescent lights. Ambient illumination was controlled by the number of these lights that were illuminated, and was defined by the down welling illumination received at the stimuli under different conditions.

At the start of a daily training or testing session each bird entered the building from an adjacent aviary. After entering the water each bird proceeded through a number of discrimination trials with each trial signalled by the opening of a guillotine gate that controlled access to the swimway (Fig. 7). When the gate was opened the bird travelled along the swimway and performed the discrimination at a known viewing distance from the gratings. Viewing distance was established by vertically dividing the runway a known distance from the stimuli, such that the bird chose to travel to either the left or right. If the birds approached the horizontal stimuli (a ‘correct’ choice) they were provided with a fish reward (a single sprat, *Sprattus sprattus*, ca 12 g). No reward was provided if the birds approached the vertical stimuli (an ‘incorrect’ choice). Upon receiving the fish or making an incorrect choice, the birds returned to the starting position. The sequence was then was repeated until the birds were satiated.

The total number of correct trials, as well as the total number of trials performed, was scored for each bird for each session. Minimum separable acuity (i.e. the narrowest stripe width at which the birds could distinguished horizontal and vertical stripes reliably) was calculated as the interpolated 75% correct performance level. Trials were conducted at six levels of ambient illumination (1.4, 0.11, 0.028, 0.011, 0.0058, 0.0012 lux; contrast = 82%, viewing distance = 2.12 m), five levels of contrast (93%, 82%, 72%, 54%, 27%; ambient illumination = 1.4 lux, viewing distance = 2.12 m), and three viewing distances (0.63, 1.05, 2.12 m, contrast = 86%, ambient illumination = 1.4 lux). Stripe width was 0.5 to 2.5 mm in 0.5 mm increments at a viewing distance of 0.63 m; 1, 2, 3, 4 and 6 mm at a viewing distance of 1.05 m; and 4 to 20 mm in 2 mm increments at a viewing distance of 2.12 m. Stripe widths were randomly ordered between successive trial days.

Data were analysed using a repeated measures ANOVA with a single fixed factor: treatment (i.e. illumination, contrast, or viewing distance), and a random factor: Bird ID. α was set at 0.05 for all tests.

All regulated procedures were performed by British Home Office licensed personnel in possession of a Personal License, and working under the auspices of a corresponding Project License, as set out in the Animals (Scientific Procedures) Act 1986.

## Supporting Information

Movie S1 Video sequence of a cormorant performing the simultaneous visual discrimination task. Initial sequence: shows the gate (B) opening at the start of a trial. The bird comes in from the left hand side of the starting area (A) and swims towards the camera positioned at the choice point (C). Middle sequence: side view of bird swimming along the middle section of the swimway. Final sequence: the bird is viewed from the gate swimming towards the pair of stimulus panel (D and E). In this instance the bird makes an incorrect choice and exits through (G) to return to the starting area for another trial.
(1.13 MB AVI)
